# The role of C-reactive protein levels on the association of physical activity with lung function in adults

**DOI:** 10.1371/journal.pone.0222578

**Published:** 2019-09-23

**Authors:** Elaine Fuertes, Anne-Elie Carsin, Vanessa Garcia-Larsen, Stefano Guerra, Isabelle Pin, Bénédicte Leynaert, Simone Accordini, Jesús Martinez-Moratalla, Josep M. Antó, Isabel Urrutia, Audrey Le Gouellec, Joachim Heinrich, Thorarinn Gislason, Rain Jõgi, Christer Janson, Debbie Jarvis, Judith Garcia-Aymerich

**Affiliations:** 1 ISGlobal, Barcelona, Spain; 2 Universitat Pompeu Fabra (UPF), Barcelona, Spain; 3 CIBER Epidemiologia y Salud Publica (CIBERESP), Barcelona, Spain; 4 National Heart and Lung Institute, Imperial College London, London, United Kingdom; 5 Program in Human Nutrition, Department of International Health, Johns Hopkins Bloomberg School of Public Health, Baltimore, United States of America; 6 Asthma and Airway Disease Research Center, University of Arizona - Tucson, Arizona, United States of America; 7 Department of Pediatrics, CHU Grenoble Alpes, Grenoble, France; 8 INSERM, Institut for Advanced Biosciences, Grenoble, France; 9 University Grenoble Alpes, Grenoble, France; 10 UMR 1152, Pathophysiology and Epidemiology of Respiratory Diseases, INSERM, Paris, France; 11 UMR 1152, University Paris Diderot Paris, Paris, France; 12 Unit of Epidemiology and Medical Statistics, Department of Diagnostics and Public Health, University of Verona, Verona, Italy; 13 Servicio de Neumologia del Complejo, Servicio de Salud de Castilla – La Mancha (SESCAM), Hospitalario Universitario de Albacete, Albacete, Spain; 14 Facultad de Medicina de Albacete, Universidad de Castilla - La Mancha, Albacete, Spain; 15 Department of Respiratory, Galdakao Hospital, Galdakao, Spain; 16 University Grenoble Alpes, CNRS, Grenoble INP, CHU Grenoble Alpes, TIMC-IMAG, Grenoble, France; 17 Institute of Epidemiology, Helmholtz Zentrum Munchen - German Research Center for Environmental Health, Munich, Germany; 18 Institute and Outpatient Clinic for Occupational, Social and Environmental Medicine, University Hospital Munich, Ludwig Maximilians University Munich, Munich, Germany; 19 Department of Respiratory Medicine and Sleep, Landspitali University Hospital Reykjavik, Reykjavik, Iceland; 20 Lung Clinic, Tartu University Hospital, Tartu, Estonia; 21 Department of Medical Sciences: Respiratory, Allergy and Sleep Research, Uppsala University, Uppsala, Sweden; 22 MRC-PHE Centre for Environment and Health, Imperial College London, London, United Kingdom; University of Maiduguri College of Medical Sciences, NIGERIA

## Abstract

**Objective:**

Regular physical activity may be associated with improved lung function via reduced systemic inflammation, although studies exploring this mechanism are rare. We evaluated the role of C-reactive protein in blood, which is a common marker of systemic inflammation, on the association of physical activity with forced expiratory volume in one second and forced vital capacity.

**Methods:**

Cross-sectional data on spirometry, C-reactive protein levels and self-reported physical activity (yes/no; ≥2 times and ≥1hr per week of vigorous physical activity) were available in the European Community Respiratory Health Survey (N = 2347 adults, 49.3% male, 28–56 years-old). A subsample was also assessed 10 years later using the International Physical Activity Questionnaire, and tertiles of Metabolic Equivalent of Task—minutes per week spent in vigorous, moderate and walking activities were calculated (N = 671, 49.6% male, 40–67 years-old). Adjusted cross-sectional mixed linear regression models and the “mediate” package in “R” were used to assess the presence of mediation.

**Results:**

Despite positive significant associations between nearly all physical activity metrics with forced expiratory volume in one second and forced vital capacity, there was no evidence that C-reactive protein levels played a role. An influence of C-reactive protein levels was only apparent in the smaller subsample when comparing the medium to low tertiles of moderate activity (mean difference [95% CIs]: 21.1ml [5.2, 41.9] for forced expiratory volume in one second and 17.3ml [2.6, 38.0] for forced vital capacity).

**Conclusions:**

In a population of adults, we found no consistent evidence that the association of physical activity with forced expiratory volume in one second or forced vital capacity is influenced by the level of C-reactive protein in blood.

## Introduction

The evidence linking regular physical activity and improved lung function is growing and appears to suggest stronger associations among current smokers [1, 2]. Reduced systemic inflammation, induced by the anti-inflammatory effects of regular long-term physical activity, has been proposed as one potential biological pathway [3], but further research into underlying mechanisms is called for [4].

Several studies have reported inverse associations between self-reported physical activity and serum C-reactive protein (CRP) levels [5,6], a commonly used systemic inflammation marker. Higher CRP levels have also been associated with lower lung function and steeper lung function decline [7, 8]. However, no study has explicitly examined whether regular physical activity leads to higher lung function via an effect on systemic inflammation.

**Fig 1:**
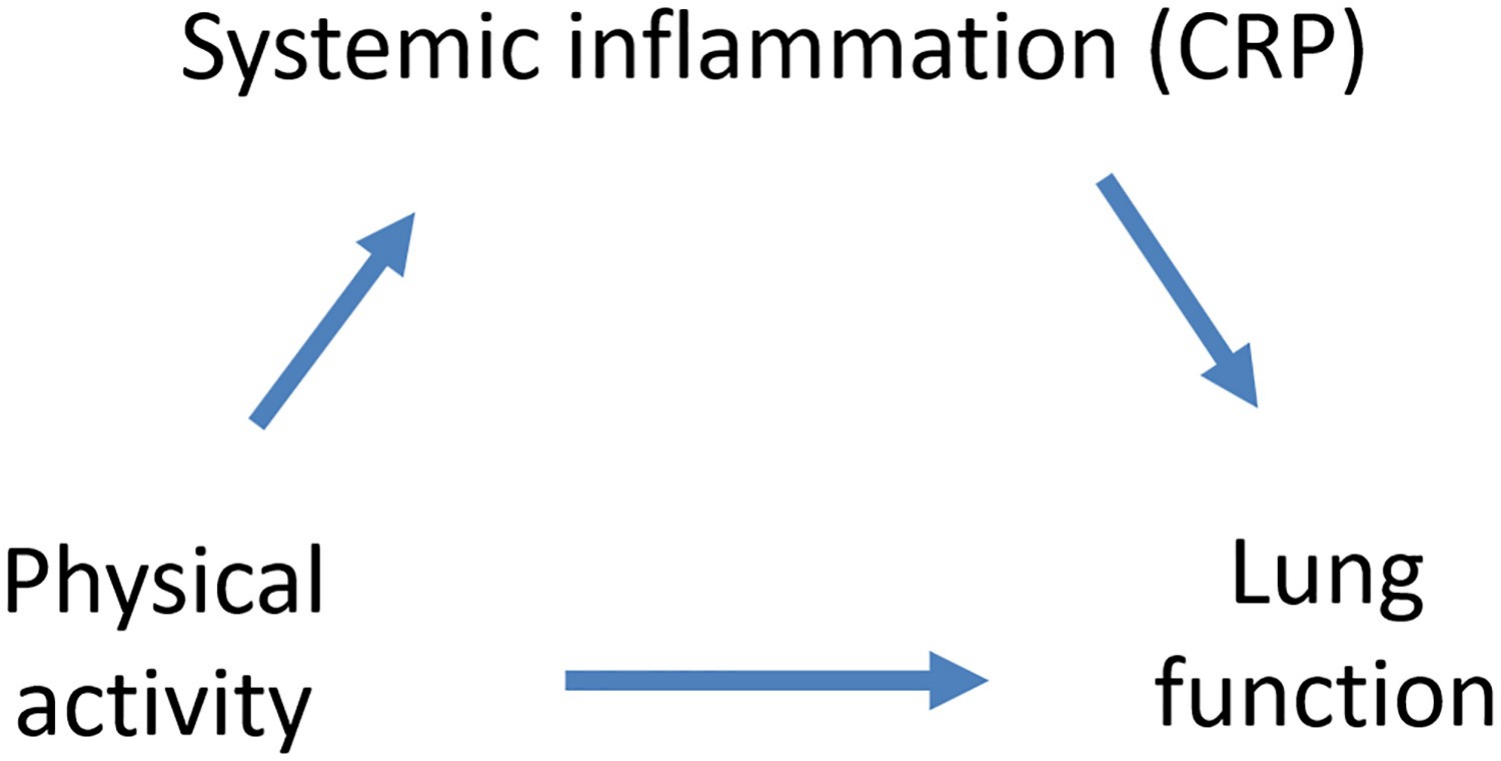
How CRP levels may influence the association between physical activity and lung function.

We previously reported that regular vigorous physical activity was positively associated with forced expiratory volume in one second (FEV_1_) and forced vital capacity (FVC) in current smokers participating in the multicentre European Community Respiratory Health Survey (ECRHS) [1]. To continue this work, we here sought to explore underlying mechanisms. Specifically, we tested the hypothetical role of C-reactive protein levels on the association of physical activity with FEV_1_ and FVC (Fig 1). Our results indicate that, despite a plausible hypothesis based on the scientific literature, there is no consistent evidence to suggest that the numerous positive associations observed between physical activity and lung function are influenced by changes in CRP levels.

## Materials and methods

### Study population

Over 18,000 participants were originally recruited into the ECRHS from 30 centers in 14 countries in 1991–1993 (ECRHS I) using population-based registers (population-based arm), with an oversampling of asthmatics (symptomatic arm) [9]. Two follow-ups approximately ten years apart have since taken place: ECRHS II in 1999–2003 and ECRHS III in 2010–2014. Extensive lifestyle and health information was collected using questionnaires and during measurements at clinical visits throughout the ECRHS (I-III). The current analysis uses cross-sectional data obtained during the first (ECRHS II) and second (ECRHS III) follow-ups, during which physical activity and spirometry data were collected [10, 11]. The analysis is limited to the eight centers that collected data on CRP levels at ECRHS II (Albacete, Barcelona, Galdakao, Grenoble, Paris, Reykjavik, Tartu and Uppsala) and the three centers that collected data on CRP levels at ECRHS III (Grenoble, Paris and Uppsala). Ethical approval from the appropriate ethics committees was obtained by all centers participating in the ECRHS and written consent was obtained from all participants.

### Lung function

Lung function without bronchodilation was assessed according to American Thoracic Society recommendations at both follow-ups [12]. FEV_1_ and FVC in absolute values (mL), repeatable to 150 mL from at least two of a maximum of five correct manoeuvres, were used in this analysis. During the lung function testing, body weight and height were measured by trained research staff.

### CRP levels

Circulating CRP was measured in frozen stored serum samples which had been taken at both follow-ups, according to standard protocols. Details of the laboratory techniques used in each country to measure the CRP levels are provided in the Supplementary Material (S1 Appendix). The CRP data were log-transformed as it followed a log-normal distribution. Afterward, as the laboratory measurement methods varied across centers, the CRP data were standardized per measurement laboratory prior to conducting the analyses (i.e. z-scores were calculated per laboratory method) to account for any potential heterogeneity. There were more participants with CRP data at the first follow-up (ECRHS II; N = 2347, main population, eight centres) compared to the second follow-up (ECRHS III; N = 671, subsample population, three centres).

### Physical activity

At ECRHS II, leisure-time vigorous physical activity data were collected using self-completed questionnaires (www.ecrhs.org/quests.htm). Participants were asked how often (frequency) and for how many hours per week (duration) they usually exercised so much that they got out of breath or sweaty. The responses for frequency were: every day, 4–6 times a week, 2–3 times a week, once a week, once a month, less than once a month and never. For statistical analyses, we grouped together the first two categories, the next two categories and the last three categories. The responses for duration were: 7 hours or more, about 4–6 hours, about 2–3 hours, about 1 hour, about half an hour and none. For statistical analyses, we grouped together the first two categories, the next two categories and the last two categories. Furthermore, as previously done in the ECRHS cohort [1,8], individuals were classified as physically active if they exercised with a frequency of at least two times per week and for a duration of at least one hour a week, and non-active otherwise.

In the subsample assessed ten years later (ECRHS III), more detailed physical activity measures were collected using the self-completed International Physical Activity Questionnaire (IPAQ-7 short form). The IPAQ collects information on time spent doing physical activity in the previous week and has been validated previously in multiple international settings and population groups [13]. Time spent in vigorous, moderate and walking activities, as well as their sum, was derived and expressed in Metabolic Equivalent of Task (METs)-min per week, following the official IPAQ scoring protocol (www.ipaq.ki.se). Tertiles of each IPAQ-variable were calculated and used in the analysis.

### Statistical analyses

To test the role of C-reactive protein levels on the association of physical activity with FEV_1_ and FVC (Fig 1), we first fit a mediator model, where CRP levels are modelled as a function of physical activity, after adjusting for relevant covariates (sex, age, height, weight, education, occupation, secondhand smoke exposure, smoking habit and a random intercept for center, which are the same as used in our previous analysis [1]). Next, we constructed the outcome model, which models lung function (the outcome) as a function of CRP levels (the mediator), including the same covariates as used in the mediator model. We used cross-sectional mixed linear regression to estimate both the mediator and outcome models.

The mediator and outcome models were then incorporated into the “mediation” package in the statistical program “R”, which estimates the amount of the association between physical activity and lung function that is occurring through changes in CRP. Specifically, the population average causal mediation effect that is occurring through the mediator (i.e. through changing CRP levels, also referred to as the indirect effect), the population average direct effect (remaining effect that is not occurring through changes in CRP levels) and the total effect (the sum of the indirect and direct effects) are calculated using previously developed algorithms [14]. Confidence intervals around these effect estimates are calculated using a quasi-Bayesian Monte Carlo method based on normal approximation, which allowed us to determine whether the estimated effects are statistically significantly different from zero (consistent with no effect). Further details of the statistical procedures have been published [15].

Sensitivity analyses included (1) stratifying by smoking (never, former, current), body mass index (< 25 kg/m^2^; 25–30 kg/m^2^; > 30 kg/m^2^) and asthma, (2) excluding weight from the models (as it could be considered a mediator itself, rather than a confounder), (3) adjusting for body mass index instead of weight, (4) dichotomizing the CRP data based on the highest quartile (≤ 75^th^ vs > 75^th^ percentile) as well as excluding the highest 5^th^ percentile of CRP levels to assess the potential influence of outliers, and finally, (5) testing for potential exposure-mediator interactions by inserting an interaction term between CRP levels (as a continuous variable) and the physical activity parameters in the “mediator model”.

Finally, we calculated E-values for the indirect, direct and total effects, which are a form of sensitivity analysis which estimates the minimum strength of association that an unmeasured confounder would need to have with both the exposure (physical activity) and the outcome (lung function), conditional on the measured covariates, to explain away any observed associations [16, 17].

## Results

Of the 3,640 participants from the eight centers that collected CRP data at ECHRS II, 2,347 had the required lung function, physical activity (i.e. activity status) and CRP information for analysis (main study population). Compared to participants from the eight centers that collected CRP information at ECRHS II but who but did not have the required data for analysis (N = 1,293, characteristics described in Table A in S1 Appendix), the 2,347 who were included were more likely to be male, older, have asthma and be in a management or technical profession.

Of the 1,097 participants from the three centers that collected CRP data at ECRHS III, 671 of those had the necessary information for analysis, including physical activity data derived using the IPAQ questionnaire (subsample). Compared to participants from the three centers that collected CRP information at ECRHS III but who but did not have the required data for analysis (N = 426, characteristics described in Table A in S1 Appendix), the 671 who were included were more likely to be in a management or technical profession.

The main (N = 2,347) and subsample (N = 671) study population characteristics are presented in Table 1. A flow chart depicting the overall derivation of the main study population and subsample is provided in Fig A in S1 Appendix.

**Table 1:** Characteristics of the study population.

Characteristic		Main study population (ECRHS II, N = 2,347)		Subsample with IPAQ data (ECRHS III, N = 671)	
		n/N or mean	% or (SD)	n/N or mean	% or (SD)
Male sex		1157/2347	49.3	333/671	49.6
Symptomatic study arm of ECRHS cohort		422/2347	18.0	82/671	12.2
Age completed full time education	< 17 years	504/2334	21.6	43/670	6.4
	17–20 years	732/2334	31.4	213/670	31.8
	> 20 years	1098/2334	47.0	414/670	61.8
Age in years^a^		42.7	(7.4)	55.3	(7.1)
Height in cm^a^		170.1	(9.8)	170.2	(9.7)
Weight in kg^a^		74.0	(15.1)	76.2	(15.6)
BMI	< 25 kg/m^2^	1188/2344	50.7	304/669	45.4
	25–30 kg/m^2^	842/2344	35.9	244/669	36.5
	> 30 kg/m^2^	314/2344	13.4	121/669	18.1
Smoking habit	Never	1000/2347	42.6	315/671	46.9
	Ex-smoker, < 15 pack-years	357/2347	15.2	140/671	20.9
	Ex-smoker, > = 15 pack-years	254/2347	10.8	118/671	17.6
	Current smoker, < 15 pack-years	279/2347	11.9	30/671	4.5
	Current smoker, > = 15 pack-years	457/2347	19.5	68/671	10.1
Secondhand smoke exposure at home or work		1000/2334	42.8	107/671	15.9
Occupation	Management/professional/non-manual	754/2347	32.1	338/671	50.4
	Technical/professional/non-manual	395/2347	16.8	155/671	23.1
	Other non-manual	549/2347	23.4	86/671	12.8
	Skilled manual	267/2347	11.4	29/671	4.3
	Semi-skilled/unskilled manual	240/2347	10.2	32/671	4.8
	Other/unknown	142/2347	6.1	31/671	4.6
Asthma		412/2343	17.6	145/670	21.6
Pre-bronchodilator FEV_1_ in ml^a^		3455.2	(810.1)	2986.5	(748.8)
Pre-bronchodilator FVC in ml^a^		4320.7	(992.4)	4000.6	(977.8)
Physically active	≥ 2 times and ≥ 1 hr per week	856/2344	36.5	--	--
Physical activity frequency	Low (< 1 a month)	976/2347	41.6	--	--
	Medium (1–3 times a week)	1016/2347	43.3	--	--
	High (> 4 times a week)	355/2347	15.1	--	--
Physical activity duration	Low (< 30 minutes)	1013/2344	43.2	--	--
	Medium (1–3 hours)	922/2344	39.3	--	--
	High (> 4 hours)	409/2344	17.4	--	--
Total physical activity (MET·min/week)	First tertile	--	--	193/611	31.6
	Second tertile	--	--	228/611	37.3
	Third tertile	--	--	190/611	31.1
Vigorous physical activity (MET·min/week)	First tertile	--	--	320/665	48.1
	Second tertile	--	--	117/665	17.6
	Third tertile	--	--	228/665	34.3
Moderate physical activity (MET·min/week)	First tertile	--	--	223/646	34.5
	Second tertile	--	--	178/646	27.6
	Third tertile	--	--	245/646	37.9
Walking physical activity (MET·min/week)	First tertile	--	--	224/633	35.4
	Second tertile	--	--	190/633	30.0
	Third tertile	--	--	219/633	34.6

BMI = body mass index; ECHRS = European Community Respiratory Health Survey; FEV_1_ = forced expiratory volume in one second; FVC = forced vital capacity; IPAQ = International Physical Activity Questionnaire; MET = Metabolic Equivalent of Task; n = number of participants with characteristic; N = total number of participants available; SD = standard deviation

^a^ The arithmetic mean and standard deviation are presented for these data which were normally distributed

There was little indication of a cross-sectional association between the physical activity parameters and continuous CRP levels (Table B in S1 Appendix). CRP was cross-sectionally associated with FEV_1_ and FVC in the main population at ECRHS II (-112.3 ml [-134.2, -90.4] and -104.3 ml [-129.4, -79.2] per 1 standard deviation increase in log CRP, respectively) and in the subsample at ECRHS III (-87.4 ml [-127.6, -47.2] and -76.1 ml [-122.7, -28.5] per 1 standard deviation increase in log CRP, respectively).

Despite positive significant associations between nearly all physical activity metrics with FEV_1_ and FVC, there was no consistent evidence for a role of CRP in this association in the entire main study population or when restricted to current smokers (Table 2).

**Table 2:** Mean difference [95% confidence intervals] in lung function (ml) per increase in self-reported physical activity in the main study population at ECRHS II (N = 2347), when restricted to current smokers (N = 736), and when weight is removed from the models.

Physical activity	Effect	Main models^a^		Current smokers ^b^		Weight removed^c^	
		FEV_1_	FVC	FEV_1_	FVC	FEV_1_	FVC
Active							
Yes vs. no	Indirect (via CRP)	-1.2 [-10.2, 7.4]	-1.7 [-10.3, 6.4]	9.0 [-12.7, 29.2]	5.7 [-14.5, 24.3]	-1.0 [-10.5, 8.3]	-1.2 [-11.4, 8.7]
	Direct (not via CRP)	50.8 [4.9, 90.0]	60.3 [8.6, 105.3]	144.3 [71.7, 224.2]	148.7 [65.2, 240.3]	51.3 [7.8, 95.0]	61.8 [12.4, 111.6]
	Total	49.5 [1.6, 91.4]	58.5 [4.4, 106.1]	153.3 [75.4, 238.9]	154.4 [68.0, 248.9]	50.3 [7.0, 95.6]	60.6 [10.6, 111.4]
Frequency							
Medium vs. low	Indirect (via CRP)	-1.0 [-11.0, 8.3]	-2.7 [-12.3, 6.0]	3.8 [-16.9, 26.4]	1.3 [-17.7, 22.1]	2.4 [-7.5, 12.3]	1.1 [-9.6, 11.6]
	Direct (not via CRP)	19.0 [-27.4, 65.5]	37.3 [-14.6, 89.3]	86.6 [9.2, 168.0]	113.6 [26.4, 205.5]	20.1 [-26.9, 61.7]	41.2 [-12.3, 89.0]
	Total	18.0 [-28.6, 65.4]	34.6 [-17.9, 87.6]	90.4 [11.1, 173.5]	114.9 [25.2, 207.0]	22.5 [-26.0, 66.5]	42.2 [-12.8, 92.2]
High vs. low	Indirect (via CRP)	-3.6 [-17.0, 9.4]	-4.3 [-17.1, 8.0]	0.5 [-29.0, 33.9]	-2.9 [-30.8, 27.3]	-7.1 [-20.9, 6.9]	-7.9 [-22.8, 7.1]
	Direct (not via CRP)	63.3 [1.1, 124.2]	71.0 [-0.5, 139.1]	126.9 [15.8, 241.5]	92.3 [-36.2, 225.5]	65.5 [4.1, 126.4]	71.9 [2.4, 141.5]
	Total	59.7 [-4.5, 120.5]	66.7 [-4.9, 135.5]	127.4 [13.6, 245.2]	89.4 [-38.2, 225.4]	58.4 [-3.8, 121.0]	64.0 [-6.7, 135.2]
Duration							
Medium vs. low	Indirect (via CRP)	-2.3 [-12.5, 7.5]	-2.5 [-12.3, 6.6]	6.8 [-14.1, 29.8]	4.6 [-14.2, 26.0]	-0.5 [-10.6, 9.6]	-0.4 [-11.1, 10.5]
	Direct (not via CRP)	41.6 [-5.1, 89.3]	51.3 [-2.2, 105.8]	152.4 [73.1, 233.1]	165.2 [75.8, 258.4]	42.5 [-3.7, 85.6]	53.9 [1.4, 103.1]
	Total	39.3 [-7.5, 87.2]	48.9 [-4.7, 103.3]	159.2 [80.4, 240.6]	169.9 [79.9, 261.5]	42.0 [-7.0, 85.7]	53.5 [-1.8, 102.9]
High vs. low	Indirect (via CRP)	-4.4 [-17.2, 7.9]	-3.8 [-15.7, 7.7]	-0.6 [-29.1, 31.6]	-3.9 [-30.0, 25.0]	-4.7 [-17.9, 8.3]	-4.5 [-18.7, 9.0]
	Direct (not via CRP)	61.2 [2.8, 118.7]	70.2 [3.8, 136.0]	142.6 [35.1, 254.8]	94.7 [-26.9, 221.6]	63.1 [5.6, 120.0]	73.3 [8.7, 138.0]
	Total	56.8 [-2.2, 113.7]	66.4 [1.0, 130.2]	141.9 [32.4, 254.1]	90.8 [-31.1, 220.9]	58.4 [0.9, 117.0]	68.8 [3.7, 135.1]

CRP = C-reactive protein; ECHRS = European Community Respiratory Health Survey; FEV_1_ = forced expiratory volume in one second; FVC = forced vital capacity

^a^ Models are adjusted for sex, age, height, weight, education, occupation, secondhand smoke exposure, smoking habit and include a random intercept for center.

^b^ Models are adjusted for sex, age, height, weight, education, occupation, secondhand smoke exposure and include a random intercept for center.

^c^ Models are adjusted for sex, age, height, education, occupation, secondhand smoke exposure, smoking habit and include a random intercept for center.

When using the IPAQ-physical activity variables available in the subsample assessed ten years later, a role of CRP on the association between physical activity levels and lung function was only apparent when comparing the second to first tertiles of METs in moderate activity (mean difference and 95% confidence intervals of 21.1 [5.2, 41.9] ml and 17.3 [2.6, 38.0] ml for FEV_1_ and FVC, respectively, Table 3).

**Table 3:** Mean difference [95% confidence intervals] in lung function (ml) per increase in self-reported physical activity in the subsample with IPAQ data at ECRHS III (N = 671), when restricted to current smokers (N = 98), and when weight is removed from the models.

Physical activity (IPAQ)	Effect	Main models^a^		Current smokers ^b^		Weight removed^c^	
		FEV_1_	FVC	FEV_1_	FVC	FEV_1_	FVC
Total							
T2 vs T1	Indirect (via CRP)	9.7 [-6.3, 27.9]	7.0 [-6.1, 23.4]	-22.0 [-93.8, 24.0]	-30.2 [-118.5, 33.7]	14.5 [-2.2, 34.3]	13.6 [-3.7, 34.6]
	Direct (not via CRP)	15.1 [-73.5, 108.7]	90.1 [-14.7, 201]	172.6 [-84.5, 453.2]	254.8 [-32.8, 519.4]	15.7 [-73.5, 108.6]	93.1 [-11.8, 203.1]
	Total	24.9 [-69.0, 120.4]	97.1 [-9.8, 209.1]	150.7 [-92.3, 426.8]	224.6 [-77.6, 486.4]	30.2 [-57.3, 122.5]	106.7 [2.4, 215.5]
T3 vs T1	Indirect (via CRP)	7.0 [-9.8, 25.8]	7.0 [-6.9, 24.6]	-11.9 [-78.1, 31.8]	-20.9 [-120.2, 47.7]	14.3 [-4.3, 33.8]	16.3 [-3.0, 38.1]
	Direct (not via CRP)	86.3 [-15.6, 180.1]	176.5 [55.8, 288.1]	284.0 [-10.6, 553.4]	274.7 [-23.9, 565.9]	91.4 [2.5, 182.1]	185.5 [79.0, 294.9]
	Total	93.3 [-7.7, 187.7]	183.5 [63.7, 296.8]	272.0 [-18.7, 539.7]	253.8 [-56.5, 563.4]	105.7 [15.1, 202.5]	201.8 [94.3, 316.7]
Vigorous							
T2 vs T1	Indirect (via CRP)	6.4 [-11.0, 24.1]	6.0 [-8.6, 23.2]	10.7 [-36.7, 80.8]	19.9 [-46.6, 115.8]	14.6 [-3.2, 35.5]	18.4 [-0.9, 42.2]
	Direct (not via CRP)	24.7 [-73.7, 131.3]	68.3 [-47.6, 193.0]	115.5 [-237.7, 479.9]	251.0 [-119.9, 639.1]	33.0 [-66.6, 139.0]	85.6 [-31.3, 209.2]
	Total	31.0 [-71.8, 140.2]	74.3 [-44.5, 199.2]	126.2 [-224.2, 491.9]	271.0 [-97.2, 669.3]	47.6 [-53.8, 151.9]	104.0 [-13.9, 225]
T3 vs T1	Indirect (via CRP)	10.4 [-2.6, 26.9]	9.5 [-1.2, 25.2]	7.8 [-22.8, 50.0]	15.9 [-21.3, 69.5]	17.5 [1.9, 35.0]	20.5 [3.4, 40.5]
	Direct (not via CRP)	100.7 [14.9, 180.0]	140.0 [38.7, 233.6]	210.4 [-18.0, 425.9]	248.0 [15.5, 462.0]	102.1 [26.3, 180.2]	147.4 [57.4, 239.4]
	Total	111.1 [25.6, 191.2]	149.5 [48.8, 243.4]	218.2 [-12.2, 429.4]	264.0 [31.9, 479.3]	119.6 [42.2, 200.8]	167.8 [76.5, 264.8]
Moderate							
T2 vs T1	Indirect (via CRP)	21.1 [5.2, 41.9]	17.3 [2.6, 38.0]	9.9 [-24.2, 62.6]	15.7 [-45.4, 87.6]	27.4 [9.7, 49.7]	30.6 [10.7, 56.2]
	Direct (not via CRP)	60.8 [-32.7, 161.2]	82.0 [-29.3, 199.3]	152.9 [-102.5, 405.6]	195.9 [-65.8, 450.0]	56.5 [-34.2, 149.8]	80.1 [-27.7, 191.6]
	Total	81.9 [-13.7, 179.7]	99.4 [-11.3, 214.0]	162.7 [-83.7, 424.5]	211.6 [-65.5, 466.3]	83.9 [-6.2, 179.4]	110.7 [2.3, 223.8]
T3 vs T1	Indirect (via CRP)	7.9 [-5.5, 24.1]	6.6 [-5.1, 21.8]	-12.3 [-72.0, 20.3]	-21.1 [-115.1, 35.5]	14.8 [-0.8, 32.3]	16.4 [-0.8, 36.4]
	Direct (not via CRP)	68.5 [-25.3, 152.2]	155.3 [43.8, 255.4]	57.1 [-212.2, 314.2]	122.9 [-151.9, 402.0]	67.6 [-12.2, 150.4]	159.0 [64.0, 257.4]
	Total	76.4 [-16.4, 162.6]	161.9 [51.6, 264.6]	44.8 [-225.0, 297.0]	101.8 [-199.0, 389.5]	82.4 [1.8, 169.7]	175.4 [79.5, 277.5]
Walking							
T2 vs T1	Indirect (via CRP)	2.5 [-14.6, 19.6]	2.2 [-12.8, 18.4]	3.8 [-26.5, 47.2]	12.9 [-38.9, 80.9]	-2.2 [-20.8, 16.1]	-2.7 [-23.2, 17.2]
	Direct (not via CRP)	-5.3 [-97.2, 92.2]	5.9 [-101.7, 119.7]	263.3 [-6.8, 536.9]	222.7 [-51.3, 502.8]	-7.2 [-97.8, 86.0]	-2.0 [-109.3, 109.1]
	Total	-2.8 [-96.9, 97.8]	8.1 [-100.3, 125.2]	267.1 [1.3, 558.2]	235.5 [-32, 533.3]	-9.4 [-99.5, 85.4]	-4.7 [-111.3, 105.5]
T3 vs T1	Indirect (via CRP)	-1.4 [-19.2, 15.8]	-2.1 [-18.7, 13.1]	-18.9 [-104, 45.5]	-50 [-161.8, 13.8]	0.9 [-18.7, 19.1]	-1.5 [-23.2, 18.2]
	Direct (not via CRP)	-1.1 [-97.6, 88.1]	-2.6 [-115.7, 102.7]	52.4 [-293.8, 363.8]	44.6 [-308.8, 359.3]	4.1 [-83.5, 94.1]	2.8 [-101.5, 108.7]
	Total	-2.5 [-97.7, 89.9]	-4.7 [-117.5, 103.1]	33.4 [-296.6, 333.3]	-5.4 [-335.3, 297.6]	5.0 [-84.3, 100.4]	1.3 [-104.4, 114.0]

CRP = C-reactive protein; ECHRS = European Community Respiratory Health Survey; FEV_1_ = forced expiratory volume in one second; FVC = forced vital capacity; IPAQ = International Physical Activity Questionnaire; T1 = first tertile; T2 = second tertile; T3 = third tertile

^a^ Models are adjusted for sex, age, height, weight, education, occupation, secondhand smoke exposure, smoking habit and include a random intercept for center.

^b^ Models are adjusted for sex, age, height, weight, education, occupation, secondhand smoke exposure and include a random intercept for center.

^c^ Models are adjusted for sex, age, height, education, occupation, secondhand smoke exposure, smoking habit and include a random intercept for center.

All listed sensitivity analyses conducted yielded similar null findings for a role of CRP, and we found no evidence to suggest the existence of potential mediator-exposure interactions. Only the removal of weight from the model led to one additional significant mediation association when comparing the third to first tertiles of METs in vigorous physical activity in the subsample (17.5 [1.9, 35.0] ml for FEV_1_ and 20.5 [3.4, 40.5] ml for FVC, Table 3).

E-values for the observed direct effects of physical activity on lung function suggest that an unmeasured confounder that was associated with both physical activity and lung function by a risk ratio of ≥ 1.30 in the main population and > 1.50 in the subsample population, after adjustment for all observed confounders, could explain away the estimate, but weaker confounding could not. For the single indirect effect observed in the subsample (when comparing the second to first tertiles of METs in moderate activity), weaker risk ratios with an unknown confounder would be required to explain away the estimates (E-values were 1.19 for FEV_1_ and 1.14 for FVC; Table C in S1 Appendix).

## Discussion

This study found no consistent evidence to suggest that the association of physical activity on lung function occurs through changes in CRP levels. Only in the subsample with more detailed physical activity data was a potential role for CRP identified for moderate physical activity, which may represent a true or chance finding. Removing weight from the models did yield one additional association as evidence for a role of CRP for vigorous physical activity in the subsample, but this result should be interpreted with caution as it is difficult to conclude whether this suggests that weight might lie in the causal pathway or that residual confounding by weight is present.

The current study was motivated by the hypothesis that regular physical activity creates an anti-inflammatory environment in the long-term [3], which leads to higher lung function. We chose to use individual-level variations in CRP levels to explore this hypothesis because studies have shown that higher physical activity is associated with lower CRP levels [5,6] and higher CRP levels are associated with both lower lung function and steeper lung function decline [7,8]. Despite this existing literature supporting our choice, CRP levels may not be the ideal marker as very high values can be observed. However, our results remained consistent in sensitivity analyses in which the CRP data were dichotomized based on the highest quartile (≤ 75^th^ vs > 75^th^ percentile) and the highest 5^th^ percentile of CRP levels were excluded.

Given our hypothesis of a role of anti-inflammation, the use of “vigorous physical activity” in the main population may thus not have been optimal, as this type of activity may be partially pro-inflammatory. However, we have previously found this type of activity to be consistently associated with higher lung function [1]. In the subsample, we did have more detailed physical activity measures available collected using the IPAQ questionnaire, which allowed more moderate anti-inflammatory types of physical activity to be considered. However, these associations risk being underpowered given the smaller sample size available.

Although we included major confounders in our analyses and tested for potential exposure-mediator interactions, we cannot verify that all assumptions were met regarding residual confounding (e.g. diet data are missing) [18]. Furthermore, participants included in this study differed from those for whom some necessary data were missing and this non-random retention may have affected the effect estimates. Notably, individuals in management or technical professions were over-represented in both the main and subsample study populations. Additionally, the directionality of any causal pathway cannot be fully assessed as all associations tested were cross-sectional due to the limited availability of repeated CRP data within centers. However, we found no indication that reverse causation may be responsible for the observed association between physical activity and lung function in a previous longitudinal analysis in the ECRHS [1].

Despite the aforementioned limitations, this study is the first to examine whether reduced systemic inflammation underlies the association between increased physical activity and improved lung function using a mediation analysis. The rather null associations observed leads us to suggest that future studies should evaluate other biomarkers of systemic inflammation, such as anti-inflammatory cytokines released from contracting skeletal muscle during exercise, and include more detailed (type and intensity) and repeated data on physical activity. Furthermore, other mechanisms should be explored, such as the potential for physical activity to lead to beneficial changes in body composition and fat distribution and changes in respiratory muscle endurance and strength, both of which may affect lung function. A better understanding of the underlying biological mechanisms is required to strengthen causal inference between physical activity and lung function. It will also provide a gateway for developing informed and effective public health policies.

## Supporting information

S1 Appendix Supplemental Material File. (DOC)

## References

[1] Fuertes E, Carsin A-E, Antó JM, Bono R, Corsico AG, Demoly P, et al Leisure-time vigorous physical activity is associated with better lung function: the prospective ECRHS study. Thorax. 2018;73(4):376–84. 10.1136/thoraxjnl-2017-210947 29306902 PMC5870462

[2] Garcia-Aymerich J, Lange P, Benet M, Schnohr P, Antó JM. Regular physical activity modifies smoking-related lung function decline and reduces risk of chronic obstructive pulmonary disease. Am J Respir Crit Care Med. 2007;175(5):458–63. 10.1164/rccm.200607-896OC 17158282

[3] Gleeson M, Bishop NC, Stensel DJ, Lindley MR, Mastana SS, Nimmo MA. The anti-inflammatory effects of exercise: mechanisms and implications for the prevention and treatment of disease. Nat Rev Immunol. 2011;11(9):607–15. 10.1038/nri3041 21818123

[4] Jenkins AR, Holden NS and Jones AW. Pulmonary rehabilitation, exercise, and exacerbations of COPD: known clinical efficacy and the unknown mechanisms. Chest. 2018;153(5):1281–82. 10.1016/j.chest.2018.01.054 29731043

[5] Kasapis C, Thompson PD. The effects of physical activity on serum C-reactive protein and inflammatory markers: a systematic review. J Am Coll Cardiol. 2005;45(10):1563–9. 10.1016/j.jacc.2004.12.077 15893167

[6] Hamer M, Sabia S, Batty GD, Shipley MJ, Tabák AG, Singh-Manoux A, et al Physical activity and inflammatory markers over 10 years: follow-up in men and women from the Whitehall II cohort study. 2012; 10.1161/CIRCULATIONAHA.112.103879 22891048 PMC3890998

[7] Fogarty AW, Jones S, Britton JR, Lewis SA, McKeever T. A prospective study of systemic inflammation and decline in lung function in a general population. Thorax. 2007;62(6):515–20. 17251312 10.1136/thx.2006.066969 PMC2117221

[8] Shaaban R, Kony S, Driss F, Leynaert B, Soussan D, Pin I, et al Change in C-reactive protein levels and FEV1 decline: A longitudinal population-based study. Respir Med. 2006;100(12):2112–20. 10.1016/j.rmed.2006.03.027 16650972

[9] Janson C, Anto J, Burney P, Chinn S, Heinrich J, Jarvis D, et al The European Community Respiratory Health Survey: what are the main results so far? Eur Respir J. 2001;18(3):598–611. 10.1183/09031936.01.00205801 11589359

[10] Burney PG, Luczynska C, Chinn S, Jarvis D. The European Community Respiratory Health Survey. Eur Respir J. 1994;7(5):954–60. 10.1183/09031936.94.07050954 8050554

[11] The European Community Respiratory Health Survey II Steering Committee. The European Community Respiratory Health Survey II. Eur Respir J. 2002;20(5):1071–9. 10.1183/09031936.02.00046802 12449157

[12] Miller M, Hankinson J, Brusasco V, Burgos F, Casaburi R, Coates A, et al Standardisation of spirometry. Eur Respir J. 2005;26:319–38. 10.1183/09031936.05.00034805 16055882

[13] Craig CL, Marshall AL, Sjorstrom M, Bauman AE, Booth ML, Ainsworth BE, et al International physical activity questionnaire:12-country reliability and validity. Med Sci Sport Exer. 2003;35:1381–95. 10.1249/01.MSS.0000078924.61453.FB 12900694

[14] Imai K, Keele L, Tingley D. A General Approach to Causal Mediation Analysis. Psychol Methods. 2010;15(4):309–334. 10.1037/a0020761 20954780

[15] Tingley D, Yamamoto T, Hirose K, Keele L and Imai K. Mediation: R package for causal mediation analysis. 2014.

[16] VanderWeele TJ, Ding P. Sensitivity analysis in observational research: introducing the E-Value. Ann Intern Med. 2017;167(4):268–274. 10.7326/M16-2607 28693043

[17] Haneuse S, VanderWeele TJ, Arterburn D. Using the E-Value to assess the potential effect of unmeasured confounding in observational studies. JAMA. 2019;321(6):602–603. 10.1001/jama.2018.21554 30676631

[18] VanderWeele T. Explanation in Causal Inference: Methods for Mediation and Interaction. Oxford University Press New York; 2015.

