# Supplementary material for: The role of C-reactive protein levels on the association of physical activity with lung function in adults
Source: PLoS One. 2019 Sep 23;14(9):e0222578. doi: 10.1371/journal.pone.0222578 (PMC6756522; doi:10.1371/journal.pone.0222578)
Supplement: S1 Appendix — (DOC) [file pone.0222578.s001.doc]

**S1 APPENDIX**

**The role of C-reactive protein levels on the association of physical activity with lung function in adults**

Elaine Fuertes^1,2,3,4^, Anne-Elie Carsin^1,2,3^, Vanessa Garcia-Larsen^5^, Stefano Guerra^1,2,3,6^, Isabelle Pin^7,8,9^, Bénédicte Leynaert^10,11^, Simone Accordini^12^, Jesús Martinez-Moratalla^13,14^, Josep M Antó^1,2,3^, Isabel Urrutia^15^, Audrey Le Gouellec^16^, Joachim Heinrich^17,18^, Thorarinn Gislason^19^, Rain Jõgi^20^, Christer Janson^21^, Debbie Jarvis^4,22^, Judith Garcia-Aymerich^1,2,3^

1. ISGlobal, Barcelona, Spain
2. Universitat Pompeu Fabra (UPF), Barcelona, Spain
3. CIBER Epidemiologia y Salud Publica (CIBERESP), Barcelona, Spain
4. National Heart and Lung Institute, Imperial College London, London, United Kingdom
5. Program in Human Nutrition, Department of International Health, Johns Hopkins Bloomberg School of Public Health - Baltimore, USA
6. Asthma and Airway Disease Research Center, University of Arizona - Tucson, Arizona, USA
7. Department of Pediatrics, CHU Grenoble Alpes, Grenoble, France
8. INSERM, Institut for Advanced Biosciences, Grenoble, France
9. University Grenoble Alpes, Grenoble, France
10. UMR 1152, Pathophysiology and Epidemiology of Respiratory Diseases, INSERM, Paris, France
11. UMR 1152, University Paris Diderot Paris, Paris, France
12. Unit of Epidemiology and Medical Statistics, Department of Diagnostics and Public Health, University of Verona, Verona, Italy
13. Servicio de Neumologia del Complejo, Servicio de Salud de Castilla – La Mancha (SESCAM), Hospitalario Universitario de Albacete, Albacete, Spain
14. Facultad de Medicina de Albacete, Universidad de Castilla - La Mancha, Albacete, Spain
15. Department of Respiratory, Galdakao Hospital, Galdakao, Spain
16. University Grenoble Alpes, CNRS, Grenoble INP, CHU Grenoble Alpes, TIMC-IMAG, Grenoble, France
17. Institute of Epidemiology, Helmholtz Zentrum Munchen - German Research Center for Environmental Health, , Munich, Germany
18. Institute and Outpatient Clinic for Occupational, Social and Environmental Medicine, University Hospital Munich, Ludwig Maximilians University Munich, Munich, Germany
19. Department of Respiratory Medicine and Sleep, Landspitali University Hospital Reykjavik, Reykjavik, Iceland
20. Lung Clinic, Tartu University Hospital, Tartu, Estonia
21. Department of Medical Sciences: Respiratory, Allergy and Sleep Research, Uppsala University, Uppsala, Sweden
22. MRC-PHE Centre for Environment and Health, Imperial College London, London, United Kingdom

**Measurement of CRP**

CRP levels at the first follow-up (ECRHS II) in the three Spanish centers (Albacete, Barcelona and Galdakao) were measured using a Millipore HNDG2MAG-36K | MILLIPLEX MAP Human Neurodegenerative Disease Magnetic Bead Panel 2 - Neuroscience Multiplex Assay. No measured samples were below the lowest standard limit. 19 samples were higher than the highest standard value and were re-coded to twice the highest standard value. The interplate coefficient of variability was 29% and the intraplate coefficient of variability was 15%.

CRP levels at the first follow-up (ECRHS II) in Reykjavik, Uppsala and Tartu were all measured at the Department of Clinical Biochemistry, Landspitali University Hospital, Iceland on a Hitachi 911 analyser using a commercially available latex enhanced immunoturbidimetric assay from Roche. The lower detection limit of the assay is 0.1 mg/l. The between-day coefficient of variation was 1.1% at a concentration of 3.7 mg/l and 1.9% at a concentration of 0.7 mg. For Uppsala, which also had CRP data available at the second follow-up (ECRHS III), a Kone 30 analyser using a commercially available latex-enhanced immunoturbidimetric assay from Roche Diagnostic Systems (Mannheim, Germany) was used. The lower detection limit of this assay is 0.1 mg/L.

CRP levels at the first follow-up (ECRHS II) in the two French centers (Grenoble and Paris) were measured at the Biochemistry Department of Bichat Hospital, Paris, by means of an ultra-sensitive competitive immunoassay on a BN II analyzer (Dade Behring, Marburg, Germany). The intra- and interassay coefficients of variation were both <10%. CRP levels was also measured at ECRHS III for these two French centers by turbidimetric assay on a SPAPlus® system (BindingSite, France), with a full range C-reactive protein kit, according to the manufacturer’s instructions. The lower detection limit of this assay is 0.05 mg/L and the method is linear up to 40mg/L.

**Table A.** **Characteristics of the participants excluded from the analyses**

|  | | **Excluded from**  **main population**  **(ECRHS II, N=1,293)** | | | **Excluded from**  **subsample population**  **(ECRHS III, N=426)** | | |
| --- | --- | --- | --- | --- | --- | --- | --- |
|  |  | **n/N or mean** | **% or (SD)** | **p-value^a^** | **n/N or mean** | **% or (SD)** | **p-value^b^** |
| Male sex |  | 568/1293 | 43.9 | 0.002 | 202/426 | 47.4 | 0.515 |
| Symptomatic study arm of ECRHS cohort | | 226/1293 | 17.5 | 0.739 | 49/426 | 11.5 | 0.793 |
| Age completed full time education | < 17 years | 274/1284 | 21.3 | 0.938 | 39/423 | 9.2 | 0.199 |
|  | 17-20 years | 398/1284 | 31.0 |  | 124/423 | 29.3 |  |
|  | > 20 years | 612/1284 | 47.4 |  | 260/423 | 61.5 |  |
| Age in years^c^ | | 41.5 | 7.4 | <0.001 | 55.4 | 7.3 | 0.960 |
| Height in cm^c^ | | 169.8 | 9.9 | 0.461 | 170.9 | 8.9 | 0.265 |
| Weight in kg^c^ | | 73.4 | 16.2 | 0.400 | 77.2 | 14.7 | 0.398 |
| BMI | < 25 kg/m^2^ | 307/572 | 53.7 | 0.365 | 97/236 | 41.1 | 0.143 |
|  | 25 - 30 kg/m^2^ | 198/572 | 34.6 |  | 103/236 | 43.6 |  |
|  | > 30 kg/m^2^ | 67/572 | 11.7 |  | 36/236 | 15.3 |  |
| Smoking habit | Never | 453/1031 | 43.9 | 0.121 | 143/303 | 47.2 | 0.863 |
|  | Ex-smoker, < 15 pack-years | 152/1031 | 14.7 |  | 57/303 | 18.8 |  |
|  | Ex-smoker, >= 15 pack-years | 87/1031 | 8.4 |  | 53/303 | 17.5 |  |
|  | Current smoker, < 15 pack-years | 145/1031 | 14.1 |  | 13/303 | 4.3 |  |
|  | Current smoker, >= 15 pack-years | 194/1031 | 18.8 |  | 37/303 | 12.2 |  |
| Secondhand smoke exposure at home or work | | 539/1286 | 41.9 | 0.612 | 72/424 | 17.0 | 0.714 |
| Occupation | Management/professional/non-manual | 409/1293 | 31.6 | <0.001 | 198/426 | 46.5 | 0.004 |
|  | Technical/professional/non-manual | 206/1293 | 15.9 |  | 73/426 | 17.1 |  |
|  | Other non-manual | 306/1293 | 23.7 |  | 68/426 | 16.0 |  |
|  | Skilled manual | 107/1293 | 8.3 |  | 23/426 | 5.4 |  |
|  | Semi-skilled/unskilled manual | 122/1293 | 9.4 |  | 24/426 | 5.6 |  |
|  | Other/unknown | 143/1293 | 11.1 |  | 40/426 | 9.4 |  |
| Asthma |  | 182/1289 | 14.1 | 0.008 | 79/426 | 18.5 | 0.245 |
| Pre-bronchodilator FEV_1_ in ml^c^ | | 3447.5 | 815 | 0.859 | 2990.3 | 706.7 | 0.956 |
| Pre-bronchodilator FVC in ml^c^ | | 4268.9 | 1010.6 | 0.337 | 4036.3 | 958.7 | 0.704 |

BMI = body mass index; ECHRS = European Community Respiratory Health Survey; FEV_1_ = forced expiratory volume in one second; FVC = forced vital capacity; n = number of participants with characteristic; N= total number of participants available; SD = standard deviation

^a^ Comparing those included (N=2,347, Table 1) and excluded (N=1,293, Table A) from the analysis at ECHRS II (Student’s t-test for normally distributed continuous variables and chi-square test for categorical variables)

^b^ Comparing those included (N=671, Table 1) and excluded (N=426, Table A) from the analysis at ECHRS III (Student’s t-test for normally distributed continuous variables and chi-square test for categorical variables)

^c^ The arithmetic mean and standard deviation are presented for these data which were normally distributed

**
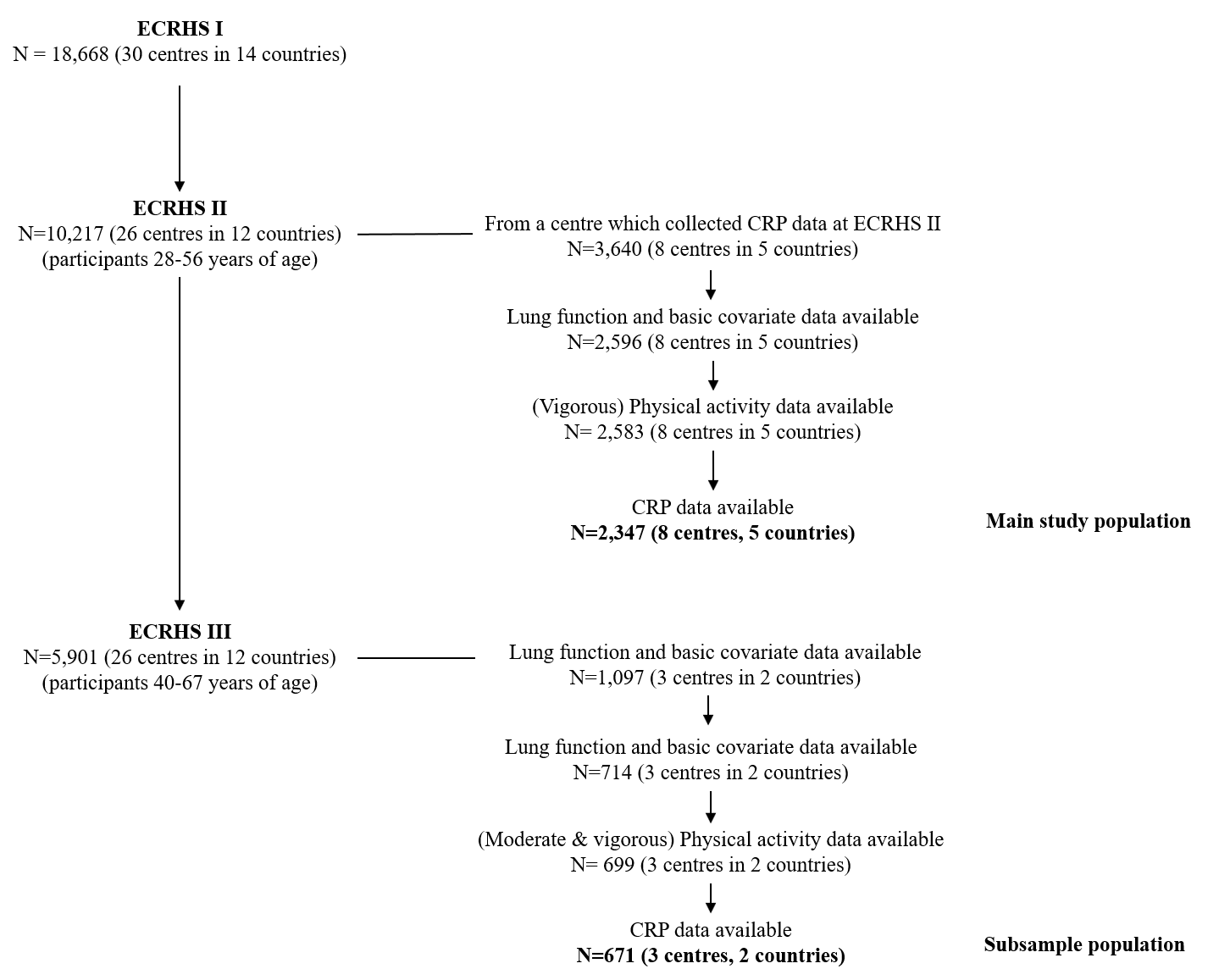
**

**Fig A. Flow chart of the main study population and the subsample with available IPAQ data**

**Table B. Cross-sectional associations between physical activity and CRP levels in the main study population and the subsample with IPAQ data.** Means ratios and their corresponding 95% confidence intervals are presented per increase in physical activity parameter^a^.

|  |  |  | **Main models^b^** | **Current smokers^c^** | **Weight removed^d^** |
| --- | --- | --- | --- | --- | --- |
| **Main study population (ECRHS II,**  **N = 2347)** | Active | no | reference | reference | reference |
|  |  | yes | 1.01 [0.94, 1.10] | 0.95 [0.82, 1.11] | 1.01 [0.93, 1.10] |
|  | Frequency | low | reference | reference | reference |
|  |  | medium | 1.01 [0.93, 1.10] | 0.98 [0.84, 1.14] | 0.98 [0.90, 1.07] |
|  |  | high | 1.04 [0.93, 1.16] | 1.01 [0.82 1.25] | 1.07 [0.95, 1.21] |
|  | Duration | low | reference | reference | reference |
|  |  | medium | 1.03 [0.94, 1.12] | 0.96 [0.83, 1.12] | 1.01 [0.92, 1.10] |
|  |  | high | 1.04 [0.94, 1.16] | 1.01 [0.82, 1.25] | 1.04 [0.93, 1.17] |
| **Subsample with IPAQ data**  **(ECRHS III, N=671)** | Total | first tertile | reference | reference | reference |
|  |  | second tertile | 0.94 [0.80, 1.11] | 1.60 [1.13, 2.26] | 0.90 [0.75, 1.09] |
|  |  | third tertile | 0.96 [0.80, 1.14] | 1.23 [0.85, 1.79] | 0.90 [0.74, 1.09] |
|  | Vigorous | first tertile | reference | reference | reference |
|  |  | second tertile | 0.90 [0.75, 1.09] | 0.98 [0.63, 1.52] | 0.83 [0.67, 1.01] |
|  |  | third tertile | 0.89 [0.77, 1.03] | 0.75 [0.55, 1.03] | 0.83 [0.71, 0.97] |
|  | Moderate | first tertile | reference | reference | reference |
|  |  | second tertile | 0.84 [0.71, 0.99] | 0.97 [0.69, 1.37] | 0.78 [0.65, 0.94] |
|  |  | third tertile | 0.94 [0.80, 1.10] | 1.39 [0.98, 1.96] | 0.89 [0.75, 1.05] |
|  | Walking | first tertile | reference | reference | reference |
|  |  | second tertile | 1.00 [0.84, 1.18] | 1.12 [0.80, 1.58] | 1.04 [0.86, 1.25] |
|  |  | third tertile | 1.02 [0.86, 1.21] | 1.46 [1.02, 2.10] | 1.02 [0.85, 1.23] |

ECHRS = European Community Respiratory Health Survey; IPAQ = International Physical Activity Questionnaire

^a^ A means ratios can be interpreted as the percentage change in the mean of the outcome variable in one group compared to the reference group.

^b^ Models are adjusted for sex, age, height, weight, education, occupation, secondhand smoke exposure, smoking habit and include a random intercept for center.

^c^ Models are adjusted for sex, age, height, weight, education, occupation, secondhand smoke exposure and include a random intercept for center.

^d^ Models are adjusted for sex, age, height, education, occupation, secondhand smoke exposure, smoking habit and include a random intercept for center.

**Table C:** E-values for the point estimate and the limit of the 95% confidence interval closest to the null^a^

| **Physical activity** | | **Effect** | **FEV_1_** | | **FVC** | |
| --- | --- | --- | --- | --- | --- | --- |
|  |  |  | **Point estimate** | **95% CI**  **closest to null** | **Point estimate** | **95% CI**  **closest to null** |
| **Main study population**  **(ECRHS II, N = 2347)** | | |  |  |  |  |
| Active | |  |  |  |  |  |
|  | Yes vs. no | Indirect (via CRP) | 1.04 | 1.00 | 1.04 | 1.00 |
|  |  | Direct (not via CRP) | 1.31 | 1.11 | 1.30 | 1.12 |
|  |  | Total | 1.30 | 1.08 | 1.30 | 1.09 |
| Frequency | |  |  |  |  |  |
|  | Medium vs. low | Indirect (via CRP) | 1.03 | 1.00 | 1.05 | 1.00 |
|  |  | Direct (not via CRP) | 1.17 | 1.00 | 1.22 | 1.00 |
|  |  | Total | 1.16 | 1.00 | 1.21 | 1.00 |
|  | High vs. low | Indirect (via CRP) | 1.07 | 1.00 | 1.07 | 1.00 |
|  |  | Direct (not via CRP) | 1.36 | 1.05 | 1.34 | 1.04 |
|  |  | Total | 1.34 | 1.00 | 1.32 | 1.00 |
| Duration | |  |  |  |  |  |
|  | Medium vs. low | Indirect (via CRP) | 1.05 | 1.00 | 1.05 | 1.00 |
|  |  | Direct (not via CRP) | 1.27 | 1.00 | 1.27 | 1.00 |
|  |  | Total | 1.26 | 1.00 | 1.27 | 1.00 |
|  | High vs. low | Indirect (via CRP) | 1.08 | 1.00 | 1.06 | 1.00 |
|  |  | Direct (not via CRP) | 1.35 | 1.07 | 1.33 | 1.07 |
|  |  | Total | 1.33 | 1.00 | 1.32 | 1.04 |
| **Subsample with IPAQ data**  **(ECRHS III, N=671)** | | |  |  |  |  |
| Total | |  |  |  |  |  |
|  | T2 vs T1 | Indirect (via CRP) | 1.12 | 1.00 | 1.09 | 1.00 |
|  |  | Direct (not via CRP) | 1.16 | 1.00 | 1.40 | 1.00 |
|  |  | Total | 1.21 | 1.00 | 1.42 | 1.00 |
|  | T3 vs T1 | Indirect (via CRP) | 1.10 | 1.00 | 1.09 | 1.00 |
|  |  | Direct (not via CRP) | 1.46 | 1.00 | 1.64 | 1.31 |
|  |  | Total | 1.49 | 1.00 | 1.66 | 1.33 |
| Vigorous | |  |  |  |  |  |
|  | T2 vs T1 | Indirect (via CRP) | 1.10 | 1.00 | 1.08 | 1.00 |
|  |  | Direct (not via CRP) | 1.21 | 1.00 | 1.33 | 1.00 |
|  |  | Total | 1.24 | 1.00 | 1.35 | 1.00 |
|  | T3 vs T1 | Indirect (via CRP) | 1.13 | 1.00 | 1.10 | 1.00 |
|  |  | Direct (not via CRP) | 1.52 | 1.17 | 1.54 | 1.25 |
|  |  | Total | 1.55 | 1.23 | 1.56 | 1.28 |
| Moderate | |  |  |  |  |  |
|  | T2 vs T1 | Indirect (via CRP) | 1.19 | 1.06 | 1.14 | 1.00 |
|  |  | Direct (not via CRP) | 1.37 | 1.00 | 1.37 | 1.00 |
|  |  | Total | 1.45 | 1.00 | 1.42 | 1.00 |
|  | T3 vs T1 | Indirect (via CRP) | 1.11 | 1.00 | 1.08 | 1.00 |
|  |  | Direct (not via CRP) | 1.40 | 1.00 | 1.58 | 1.27 |
|  |  | Total | 1.43 | 1.00 | 1.60 | 1.29 |
| Walking | |  |  |  |  |  |
|  | T2 vs T1 | Indirect (via CRP) | 1.06 | 1.00 | 1.05 | 1.00 |
|  |  | Direct (not via CRP) | 1.09 | 1.00 | 1.08 | 1.00 |
|  |  | Total | 1.06 | 1.00 | 1.09 | 1.00 |
|  | T3 vs T1 | Indirect (via CRP) | 1.04 | 1.00 | 1.05 | 1.00 |
|  |  | Direct (not via CRP) | 1.04 | 1.00 | 1.05 | 1.00 |
|  |  | Total | 1.06 | 1.00 | 1.07 | 1.00 |

CRP = C-reactive protein; ECHRS = European Community Respiratory Health Survey; FEV_1_ = forced expiratory volume in one second; FVC = forced vital capacity IPAQ = International Physical Activity Questionnaire; T1 = first tertile; T2 = second tertile; T3 = third tertile

^a^ Calculated according to [1].

**Local Principal Investigators, senior scientific teams and funding agencies for the participating European Community Respiratory Health Survey centers**

**ECRHS II Principal Investigators and senior scientific teams**

**France: Paris** (F. Neukirch, B. Leynaert, R. Liard, M. Zureik), **Grenoble** (I. Pin, J. Ferran-Quentin); **Spain: Albacete** (J. Martinez-Moratalla Rovira, E. Almar, M. Arévalo, C. Boix, G González, J.M. Ignacio García, J. Solera, J Damián), **Galdakao** (N. Muñozguren, J. Ramos, I. Urrutia, U. Aguirre), **Barcelona** (J. M. Antó, J. Sunyer, M. Kogevinas, J. P. Zock, X. Basagaña, A. Jaen, F. Burgos, C. Acosta); **Sweden**: **Uppsala** (C. Janson, G. Boman, D. Norback, G. Wieslander, M. Gunnbjornsdottir); **Iceland: Reykjavik** (T. Gislason, E. Bjornsson, D. Gislason, K.B Jörundsdóttir*);* **Estonia: Tartu** (R. Jõgi, A. Soon);

**Financial Support for ECRHS II**

**France: (All)** Programme Hospitalier de Recherche Clinique—Direction de la Recherche Clinique (DRC) de Grenoble 2000 number 2610, Ministry of Health, Ministère de l’Emploi et de la Solidarité, Direction Génerale de la Santé, Centre Hospitalier Universitaire (CHU) de Grenoble, **Grenoble:** Comite des Maladies Respiratoires de l’Isere, **Paris:** Union Chimique Belge- Pharma (France), Aventis (France), Glaxo France; **Spain: Albacete:** Fondo de Investigacion Santarias (grant codes, 97/0035-01,99/0034-01 and 99/0034 02), HospitalUniversitario de Albacete, Consejeria de Sanidad, **Barcelona:** Sociedad Espanola de Neumologı´a y Cirugı´a Toracica, Public Health Service(grant code, R01 HL62633-01), Fondo de Investigaciones Santarias (grant codes, 97/0035-01, 99/0034-01, and 99/0034-02), Consell Interdepartamentalde Recerca i Innovacio´ Tecnolo`gica (grant code, 1999SGR 00241) Instituto de Salud Carlos III; Red deCentros de Epidemiologı´a y Salud Pu´blica, C03/09,Redde Basesmoleculares y fisiolo´gicas de lasEnfermedadesRespiratorias,C03/011and Red de Grupos Infancia y Medio Ambiente G03/176, **Galdakao:** Basque Health Department; **Sweden: Uppsala**: Swedish Heart Lung Foundation, Swedish Foundation for Health Care Sciences and Allergy Research, Swedish Asthma and Allergy Foundation, Swedish Cancer and Allergy Foundation, Swedish Council for Working Life and Social Research (FAS); **Iceland: Reykjavik:** Icelandic Research Council, Icelandic University Hospital Fund; **Estonia: Tartu** Estonian Science Foundation grant no 4350;

**ECRHS III Principal Investigators and senior scientific teams**

**France: Grenoble** (I. Pin, V. Siroux, J.Ferran, J.L Cracowski), **Paris** (B. Leynaert, D. Soussan, D. Courbon, C. Neukirch, L. Alavoine, X. Duval, I. Poirier); **Sweden: Uppsala:**(B Andersson, D Norback, U Spetz Nystrom, G Wieslander, G.M Bodinaa Lund, K Nisser).

**Financial Support for ECRHS III**

**France:** (**All**) Ministère de la Santé. Programme Hospitalier de Recherche Clinique (PHRC) national 2010, **Grenoble:** Comite Scientifique AGIRadom 2011, **Paris:** Agence Nationale de la Santé, Région Ile de France, domaine d’intérêt majeur (DIM); **Sweden: Uppsala:** The Swedish Heart and Lung Foundation, The Swedish Asthma and Allergy Association, The Swedish Association against Lung and Heart Disease, Swedish Research Council for health, working life and welfare (FORTE).

**References**

1. VanderWeele TJ, Ding P. Sensitivity analysis in observational research: introducing the E-Value. Ann Intern Med. 2017;167(4):268-274. doi:10.7326/M16-2607
